# Mind the size: LARGE3-OsHDT1 complex keeps rice grain size in line

**DOI:** 10.1093/plcell/koag117

**Published:** 2026-04-22

**Authors:** Leonardo I Pereyra-Bistraín

**Affiliations:** Assistant Features Editor, The Plant Cell, American Society of Plant Biologists; Institute of Plant Sciences Paris-Saclay (IPS2), Université Paris-Saclay, CNRS, INRAE, Univ Evry, Orsay 91405, France; Institute of Plant Sciences Paris-Saclay (IPS2), Université de Paris Cité, Gif-sur-Yvette F-91190, France

Grain size is one of the most important traits in cereal crops. Just consider maize: its wild ancestor, teosinte, went from a grass with a few small, hard, scattered grains to modern maize, boasting hundreds of large, edible grains. Deciphering how grain size is controlled is therefore essential both for plant developmental biology and agriculture.

Over the past decades, scientists have shown that grain size is not governed by just a few genes; instead, it is a trait determined by multiple genes. These genes function in several cell signaling pathways, including phytohormone signaling, transcriptional regulation, and the ubiquitin-proteasome, among others (Gasparis and Miłoszewski 2023). Despite substantial progress in deciphering the mechanisms controlling grain size, the extent to which epigenetic processes shape this trait remains an open question.

Previous studies indicate that changes in histone acetylation can affect grain size (Huang et al. 2024; Shen et al. 2024), yet how this mechanism is directed toward specific gene targets remains to be elucidated. In addressing this question, Guozheng Zhang and colleagues (Zhang et al. 2026) conducted a systematic genetic analysis in rice and identified the histone-binding protein LARGE3, which interacts with the rice histone deacetylase OsHDT1 and consequently regulates grain size. This regulation occurs by modulating histone H4 acetylation at the *OsMKKK10* gene, which encodes an upstream kinase of the mitogen-activated protein kinase (MAPK) signaling pathways.

In this recent study, the authors identified a mutant line (*large3-1*) from an ethyl methane sulfonate–treated population of the rice *japonica* variety Xiushui09, displaying a notably large and heavy-grain phenotype (Fig. 1a). Genome sequencing of the *large3-1* mutant revealed a single nucleotide G deletion at the LOC_Os01g37790 locus, suggesting that this mutation may be responsible for the phenotype. These findings were further confirmed by the functional complementation of *large3-1* with the full genomic sequence LOC_Os01g37790 (*LARGE3*), restoring grain size. Moreover, a set of clustered regularly interspaced short palindromic repeats (CRISPR)/CRISPR-associated nuclease 9 (Cas9) knockout lines, *large3-cri,* targeting the *LARGE3* gene and generated in the Zhonghua11 rice variety (ZH11), additionally confirmed the large-grain size phenotype. Interestingly, the authors found that the *large3-1* phenotype is due to an increase in cell number in the spikelet hull, indicating that LARGE3 may restrict grain size by modulating cell proliferation.

**Figure 1:**
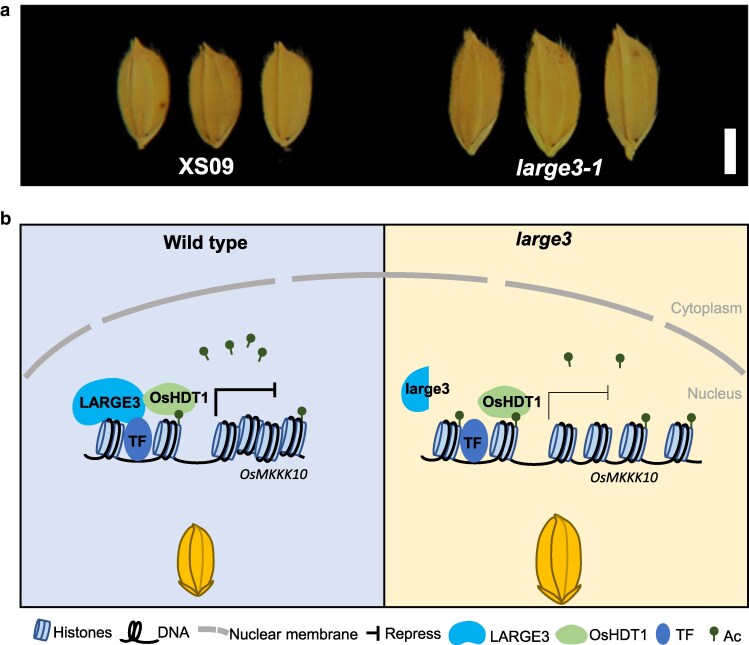
(a) LARGE3-OsHDT1 in grain size control. Rice grains of the rice *japonica* variety *Xiushui09* (XS09) and *large3-1* mutant. Bar = 2 mm. (b) Working model: LARGE3-OsHDT1 regulates grain size by controlling histone H4 acetylation leading to a dense chromatin state in *OsMKKK10* locus. Adapted from https://doi.org/10.1093/plcell/koag109, Figures 1 and 7.

Based on complementary protein interaction assays in vitro and in planta, Guozheng Zhang and colleagues (Zhang et al. 2026) characterized LARGE3 and first found that it is a nuclear protein that binds histones—predominantly H3, H4, and H2A—through its PWWP-binding domain. Second, a set of protein-DNA binding assays demonstrated that LARGE3 can also bind DNA in a nonspecific manner. Third, LARGE3 harbors a LXXLL motif responsible for binding to OsHDT1. This last point is particularly important, as *Oshdt1* mutant lines, generated by CRISPR/Cas9 in ZH11, formed longer and narrower grains. This shows that *Oshdt1* affects cell number in spikelet hulls, as occurs in *large3-1* mutants. These findings raised a key question: how does the LARGE3–OsHDT1 interaction influence grain size?

As previously shown, several members of the MAPK signaling pathway can either promote or restrict grain size (Xu et al. 2018a, 2018b). Thus, the authors hypothesized that LARGE3-OsHDT1 may regulate genes involved in the MAPK kinase pathway. By analyzing the gene expression levels of several grain-size-related genes, they found a significant gene expression increase of the *OsMKKK10* gene in the *large3-cri* and *Oshdt1-cri* mutants. Using chromatin immunoprecipitation followed by qPCR (ChIP-qPCR) in the *large3-cri1;gLARGE3-GFP* complemented line and ZH11 protoplasts, the authors successfully demonstrated that both LARGE3 and OsHDT1 can associate with the *OsMKKK10* promoter. More importantly, they can mutually enhance their association with the promoter and subsequently promote its repression.

Through a similar ChIP-qPCR approach, the study shows that H4 acetylation levels at the *OsMKKK10* gene were increased in the *large3-cri1* and *Oshdt1-cri1* mutants. Chromatin accessibility assays further demonstrate that the *OsMKKK10* gene region was ectopically open. This indicates that the LARGE3-OsHDT1 complex promotes a condensed chromatin state by facilitating H4 deacetylation, thereby restricting *OsMKKK10* expression. Interestingly, mutants generated using CRISPR/Cas9, *Osmkkk10-cri,* produced small grains, whereas the *large3-cri1 Osmkkk10-cri* double mutant exhibited an intermediate grain size compared to ZH11, revealing that LARGE3 and OsMKKK10 work, at least partially, through a common pathway to regulate grain size.

Overall, this extensive work reveals a mechanism by which the LARGE3-OsHDT1 complex modulates histone acetylation to control grain size through MAPK signaling (Fig. 1b). Future studies will help determine whether LARGE3 regulates other grain size-related genes and the mechanisms involved.

## Recent related articles in The Plant Cell

- Zhang et al. (2024) identified that the histone acetyltransferase GCN5 interacts with the transcription factor CAMTA2 and modulates wheat grain size and weight by activating starch genes via histone acetylation.
- Zhang et al. (2026) demonstrated in Arabidopsis that TCP transcription factors are key for controlling seed size by promoting endosperm cell development while restricting seed coat growth through gene repression.

## Data Availability

None to declare.
